# Interpreting Ring Currents from Hückel-Guided σ- and π-Electron Delocalization in Small Boron Rings

**DOI:** 10.3390/molecules30173566

**Published:** 2025-08-31

**Authors:** Dumer S. Sacanamboy, Williams García-Argote, Rodolfo Pumachagua-Huertas, Carlos Cárdenas, Luis Leyva-Parra, Lina Ruiz, William Tiznado

**Affiliations:** 1 Doctorado en Fisicoquímica Molecular, Facultad de Ciencias Exactas, Universidad Andrés Bello, República 275, Santiago 837014, Chile; d.sacanamboypapamija@uandresbello.edu; 2 Centro de Investigación para el Diseño de Materiales (CEDEM), Facultad de Ciencias Exactas, Departamento de Ciencias Químicas, Universidad Andrés Bello, Avenida República 275, Santiago 837014, Chile; w.garcaargote@uandresbello.edu (W.G.-A.); luis.leyva@unab.cl (L.L.-P.); 3 Laboratorio de Investigación en Química Teórica, Escuela Profesional de Química, Facultad de Ciencias Naturales y Matemáticas, Universidad Nacional Federico Villarreal, Jr. Río Chepén 290, El Agustino, Lima 15001, Peru; rpumachagua@gmail.com; 4 Departamento de Física, Facultad de Ciencias, Universidad de Chile, Av. Las Palmeras 3425, Ñuñoa, Santiago 837014, Chile; cardena@uchile.cl; 5 Center for Development of Nanoscience and Nanotechnology (CEDENNA), Av. Libertador Bernardo O Higgins 3363, Santiago 837014, Chile; 6 Institute of Biomedical Sciences, Faculty of Health Sciences, Universidad Autónoma de Chile, Santiago 8910060, Chile

**Keywords:** boron clusters, aromaticity, magnetically induced current density, AdNDP, EDDB analysis

## Abstract

The aromaticity of small boron clusters remains under scrutiny due to persistent inconsistencies between magnetic and electronic descriptors. Here, we reexamine B_3_^−^, B_3_^+^, B_4_, B_4_^2+^, and B_4_^2−^ using a multidimensional approach that integrates Adaptive Natural Density Partitioning, Electron Density of Delocalized Bonds, magnetically induced current density, and the z-component of the induced magnetic field. We introduce a model in which σ-aromaticity arises from two distinct delocalization topologies: a radial 2e^−^ σ-pathway and a tangential multicenter circuit formed by alternating filled and vacant sp^2^ orbitals. This framework accounts for the evolution of aromaticity upon oxidation or reduction, preserving coherence between electronic structure and magnetic response. B_3_^−^ features cooperative radial and tangential σ-delocalization, together with a delocalized 2e^−^ π-bond, yielding robust double aromaticity. B_3_^+^ retains σ- and π-aromaticity, but only via a tangential 6e^−^ σ-framework, leading to a more compact delocalization and slightly attenuated ring currents. In B_4_, the presence of a radial 2e^−^ σ-bond and a 4c–2e π-bond confers partial aromatic character, while the tangential 8e^−^ σ-framework satisfies the 4n rule and induces a paratropic current. In contrast, B_4_^2+^ lacks the radial σ-component but retains a tangential 8e^−^ σ-circuit and a 2e^−^ 4c–2e π-bond, leading to a σ-antiaromatic and π-aromatic configuration. Finally, B_4_^2−^, exhibits delocalized π- and σ-circuits, yielding consistent diatropic ring currents, which confirms its fully doubly aromatic nature. Altogether, this analysis underscores the importance of resolving σ-framework topology and demonstrates that, when radial and tangential contributions are correctly distinguished, Hückel’s rule remains a powerful tool for interpreting aromaticity in small boron rings.

## 1. Introduction

The concept of aromaticity, initially introduced to rationalize the remarkable stability of benzene and related compounds, has evolved into a broad and nuanced framework that encompasses organic, inorganic, and organometallic chemistry [1,2,3,4]. While classical aromaticity is rooted in π-electron delocalization within planar monocyclic systems obeying Hückel’s 4n + 2 rule, modern interpretations now include σ-, δ-, and even φ-aromaticity, each defined by the symmetry and nature of the delocalized orbitals [5,6,7]. σ-Aromaticity, for instance, plays a central role in electron-deficient systems such as planar boron clusters [8,9,10,11,12,13,14]. In contrast, δ- and φ-aromaticity involve d- and f-orbital contributions, respectively, with φ-aromaticity emerging in actinide-containing clusters featuring delocalization through f-orbitals with high angular momentum [5,6,15,16]. Small clusters—particularly those composed of main-group elements like boron—serve as ideal platforms for exploring these exotic manifestations [8,10,11,13,14,17]. These systems often exhibit multiple aromaticity, where different delocalized frameworks (e.g., σ and π) coexist independently, or even conflicting aromaticity, in which the aromatic character of one delocalized circuit opposes that of another, leading to complex magnetic or energetic responses [8,10,11,14,18]. Such phenomena challenge traditional definitions and underscore the need for a multidimensional approach to aromaticity in cluster chemistry.

Aromaticity, despite its widespread use, remains a non-observable and context-dependent concept, requiring a comprehensive evaluation using multiple, complementary criteria [18,19,20,21,22]. The energetic criterion evaluates the stabilization associated with electron delocalization, often estimated using isomerization stabilization energy (ISE) schemes [23], homodesmotic reactions [24], or block-localized wavefunction (BLW) methods [25]. In particular, the magnetic response has long played a central role, both theoretically and experimentally. The presence of magnetically induced ring currents—first inferred from the anomalously high diamagnetic susceptibility of aromatic molecules—remains a hallmark of delocalized electron systems [18,19,20,26]. Experimentally, this effect is probed through measurements such as NMR chemical shifts, which reflect the local magnetic shielding generated by these currents, and magnetic susceptibility exaltation, which captures their global influence [26,27]. This phenomenon, bridging experimental observables and theoretical models, is now routinely explored using quantum chemical methods to map current density patterns and quantify magnetic responses [27,28,29]. This is assessed computationally through nucleus-independent chemical shifts (NICS) [26], anisotropy of the induced current density (ACID), [28] magnetically induced current density (MICD) analysis [30,31], and the $B_{z}^{ind}$ index, which quantifies the out-of-plane component of the induced magnetic field at the ring center and serves as a localized probe of aromatic character [32]. The structural criterion is traditionally evaluated by bond-length equalization, with indices such as the harmonic oscillator model of aromaticity (HOMA) [33]; however, such measures may be unreliable for metallic or non-classical systems. Finally, the electronic delocalization criterion offers a direct view of aromatic bonding patterns through approaches such as Adaptive Natural Density Partitioning (AdNDP) [8], which reveals multicenter bonds, and the Electron Density of Delocalized Bonds (EDDB) [34], which quantifies both local and global delocalization based on the electron density. In small main-group clusters—especially those involving boron—the integration of these criteria is critical, as classical indicators may diverge or offer contradictory interpretations. A recent study on B_3_ clusters has emphasized the need for a multidimensional evaluation combining bonding and magnetic criteria [35].

**Figure 1:**
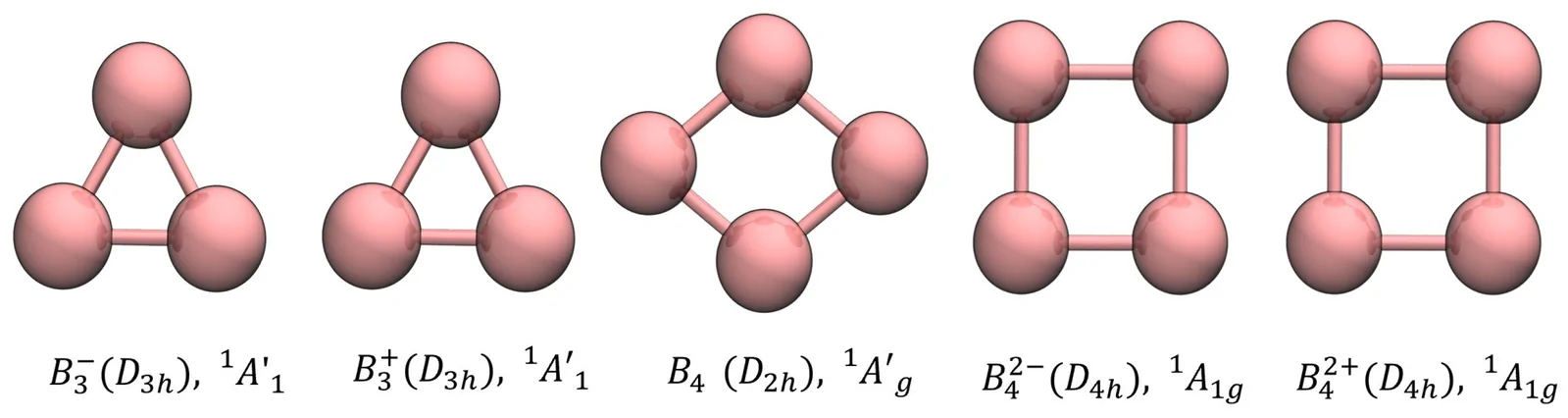
Optimized geometries of selected global minimum boron clusters at the PBE0-D3/def2-TZVP level. Point group symmetries and electronic ground states are indicated.

In this work, we present a systematic re-evaluation of aromaticity in a representative series of small, bare boron clusters (Figure 1) using state-of-the-art computational methods: EDDB [34], AdNDP [8], MICD [29,31], and the $B_{z}^{ind}$ index [32]. Our results show that several previous aromaticity assignments—particularly those relying primarily on isotropic NICS values at the ring center or above the molecular plane—can be misleading due to contamination from local, non-aromatic contributions. Notably, earlier models for planar boron clusters have invoked the particle-on-a-disk approach to classify molecular orbitals by radial and azimuthal nodal patterns or adopted the σ-radial/σ-tangential partitioning scheme originally developed for all-metal clusters, where only a subset of σ-orbitals was analyzed for convenience [36,37,38]. In contrast, our analysis separates the σ-valence electrons consistently into radial and tangential components, based on AdNDP results, when applying Hückel counts, and evaluates their individual contributions alongside the π-system. When aromaticity is assessed with complementary probes of delocalization, multicenter bonding, and magnetically induced currents, a coherent picture emerges in which simple electron counts (4n + 2 or 4n) are validated, provided they are interpreted judiciously within this multidimensional framework.

## 2. Computational Details

The geometry optimizations of the systems depicted in Figure 1 were conducted at the PBE0 [39]-D3 [40]/def2-TZVP [41] level using the Gaussian 16 program [42]. These clusters correspond to previously reported global minima and were reoptimized to ensure methodological consistency. Harmonic vibrational frequency calculations confirmed that all reoptimized structures are true minima on the potential energy surface (i.e., no imaginary frequencies).

Aromaticity was assessed using a set of complementary descriptors. Bonding analysis was performed with the AdNDP method via Multiwfn 3.8 [43], enabling the identification of localized and multicenter delocalized bonding patterns in both σ and π frameworks. The degree of electron delocalization was further quantified using the EDDB method [34], with σ- and π-resolved delocalization values extracted using the RunEDDB suite. All visualizations of molecular structures and orbitals were rendered using VMD 1.9.3 [44]. Complementarily, Intrinsic Bond Orbitals (IBOs) [45,46] were computed at the PBE0 [39]/def2-TZVP [41] level using ORCA 5.0 [47]. The localization was performed via the Pipek–Mezey scheme on Intrinsic Atomic Orbitals, yielding chemically intuitive σ- and π-bonding patterns without predefined assumptions. IBOs were visualized using IBOview [48].

Magnetic criteria of aromaticity were evaluated through the MICD, computed using the SYSMOIC program [49]. Perturbed and unperturbed molecular orbitals were obtained from Gaussian 16 using the CSGT [50] scheme at the BHandHLYP [51]/def2-TZVP [41]//PBE0-D3/def2-TZVP level. These orbitals were used as input for SYSMOIC, which applies the CTOCD-DZ method to compute gauge-origin-independent current densities under an external magnetic field perpendicular to the molecular plane. To quantify the ring current flow intensity, we evaluated the net bond current strengths. These are computed by integrating the normal component of the current density over topologically defined domains on planes perpendicular to selected internuclear axes. Each integration domain is defined by a near-zero isoline enclosing a local extremum in the modulus of the induced current density on a plane perpendicular to the selected atomic pair. The net bond current strength, expressed in nA·T^−1^, is obtained by integrating the normal component of the current density over these domains, providing a quantitative measure of electron delocalization through the selected bond [49].

Additionally, the out-of-plane component of the induced magnetic field, $B_{z}^{ind}$ [32], was calculated using Multiwfn [43]. This scalar field, evaluated 1.0 Å above the molecular plane, provides spatial information on magnetic shielding (diatropic, negative $B_{z}^{ind}$) and deshielding (paratropic, positive $B_{z}^{ind}$) regions. Contour maps and isosurfaces of $B_{z}^{ind}$ were analyzed to complement the MICD analysis. Together, these magnetic descriptors enable a robust characterization of aromaticity beyond conventional approaches.

## 3. Results and Discussion

We begin our discussion by briefly describing the set of boron clusters analyzed in this study, which includes reexamine B_3_^−^, B_3_^+^, B_4_, B_4_^2+^, and B_4_^2−^. All structures correspond to previously reported global minima [8,10,11,14]. A summary of current consensus regarding their aromatic or antiaromatic character is presented in Table 1, where we compare the reported NICS values [10,52] and electron counts derived from canonical molecular orbital (CMO) occupations, with our detailed analysis of MICD, specifically through the evaluation of dissected σ and π ring current strengths (RCSs). As shown, notable discrepancies emerge in some cases, highlighting the limitations of NICS and the importance of more rigorous analysis of the magnetic response and its relationship with the aromaticity phenomenon. In the following sections, we will discuss each system individually, examining its bonding patterns, current pathways, and delocalization features to provide a comprehensive re-evaluation of its aromatic character.

To systematically reassess the aromatic character of the selected boron clusters, we begin by evaluating their electronic structure in the framework of Hückel’s rule, which, in the context of clusters, can be extended beyond π-delocalization to include σ-delocalized bonding frameworks. The AdNDP method is employed to characterize bonding patterns in terms of localized (2c–2e) and delocalized (nc–2e) elements, allowing for a direct assessment of (4n + 2 or 4n) electron delocalization in both σ and π manifolds. We then analyze electron delocalization more quantitatively using the EDDB method, which yields delocalization indices and electron delocalization counts in the respective subspaces. Finally, we examine the magnetic response of these clusters through MICD analysis and the evaluation of the out-of-plane component of the induced magnetic field $B_{z}^{ind}.$ This integrative approach enables a coherent and multidimensional interpretation of aromaticity, grounded in electronic structure, electron delocalization, and magnetic response.

### 3.1. B_3_^−^ and B_3_^+^

**Figure 2:**
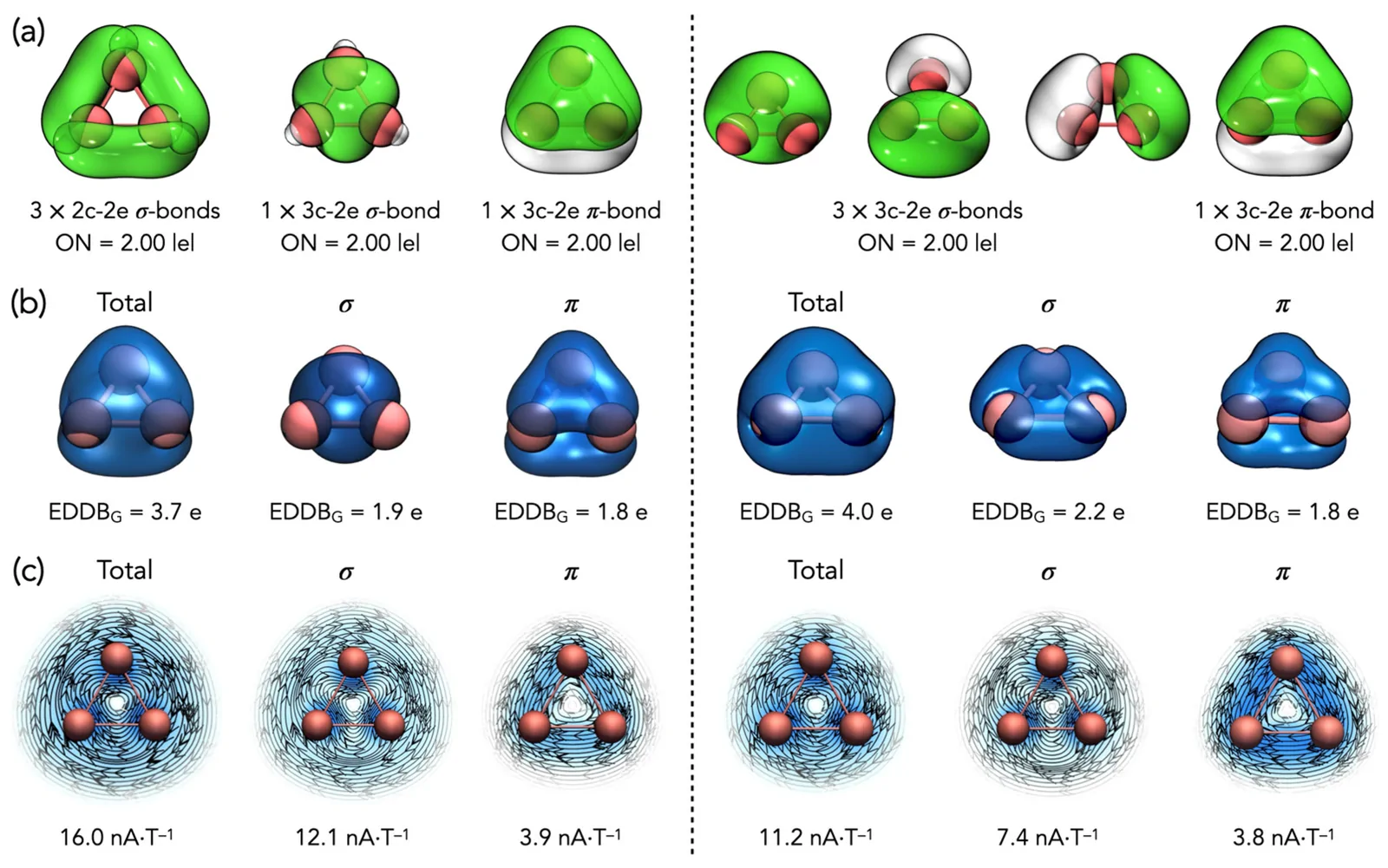
Bonding and aromaticity analysis for B_3_^−^ (**left**) and B_3_^+^ (**right**). (**a**) AdNDP-derived bonding pattern showing localized and delocalized bonds; occupation numbers (ON) are given in |e| (isovalue = ±0.05). (**b**) EDDB_G_ isosurfaces depicting total, σ and π electron delocalization, with associated electron populations in |e| (isovalue = ±0.001). (**c**) Total, σ- and π-MICD maps 0.5 Å above the molecular plane, with corresponding RCS values (in nA·T^−1^).

Previous studies based on canonical molecular orbital analysis and NICS calculations have established that the B_3_^−^ cluster exhibits double aromaticity, with both σ- and π-aromatic character consistent with the Hückel 4n + 2 rule for n = 0 [35,52,55]. In the present work, we employed the AdNDP method to elucidate the bonding framework further (Figure 2a, left). As shown in Figure 2a, the analysis recovers three classical two-center two-electron (2c–2e) σ bonds (ON = 2.00 |e|) and identifies two delocalized three-center two-electron (3c–2e) bonds: one σ-type (in-plane, often described as a radial σ bond), and one π-type, both with occupation numbers of 2.00 |e|. These delocalized bonding elements support the interpretation of B_3_^−^ as a doubly aromatic system, in agreement with earlier conclusions based on MO topology and magnetic criteria.

To further probe aromatic delocalization, we applied the EDDB method (Figure 2b, left), which quantifies electron delocalization in both σ and π frameworks. For B_3_^−^, the total EDDB value is 3.7 |e|, with comparable σ (1.9 |e|) and π (1.8 |e|) contributions, supporting its double aromatic character. As shown in Figure 2b, the σ-isosurface forms an in-plane triangular loop, while the π-isosurface adopts a toroidal shape above and below the molecular plane.

How about the magnetic response? Figure 2c displays the vector plots of the MICD computed 0.5 Å above the B_3_^−^ ring. Diatropic ring currents are observed both inside and outside the ring, with the σ component (12.1 nA·T^−^^1^) contributing more strongly than the π component (3.9 nA·T^−^^1^), yielding a total RCS of 16.0 nA·T^−^^1^. This value exceeds that of benzene (12.2 nA·T^−^^1^ at the same level), indicating a pronounced aromatic character. This σ-dominated magnetic response contrasts with the nearly balanced σ/π electron delocalization predicted by EDDB, as well as the two σ- and two π-delocalized electrons recovered from the AdNDP analysis, suggesting that magnetic and electronic descriptors may emphasize different aspects of aromaticity. Figure S1 shows the $B_{z}^{ind}$ isosurfaces and planes. The −3.00 ppm isosurface (left), along with the contour maps in the molecular plane (center) and in a perpendicular plane through the ring (right), reveals intense shielding within and around the ring, consistent with long-range diatropic behavior and further supporting the system’s aromatic character.

To understand how oxidation influences aromaticity, we compare B_3_^−^ and B_3_^+^. The loss of two electrons in B_3_^+^ could affect either the σ- or π-frameworks, and previous interpretations based on CMOs and NICS analyses have suggested a disruption of the σ-system, leading to a π-only aromatic character. However, our AdNDP analysis reveals a fully delocalized bonding topology in B_3_^+^ (Figure 2a, right), with three 3c–2e σ-bonds and one 3c–2e π-bond (ON = 2.00 |e|), while an alternative analysis recovers three 2c–2e σ-bonds with ON ≈ 1.76 |e| (Figure S2). Thus, it is best described as a 6-electron (4n + 2) tangential circuit. EDDB further supports this dual (σ and π) aromatic character, with 2.2 |e| from σ-contributions and 1.8 |e| from π-contributions (Figure 2b, right). MICD analysis reveals coexisting σ and π diatropic ring currents of 7.4 and 3.8 nA·T^−^^1^, respectively (total: 11.2 nA·T^−^^1^ (Figure 2c, right)). This magnetic behavior is further corroborated by $B_{z}^{ind}$ isosurfaces and planes (Figure S3), which reveal shielding topologies analogous to those of B_3_^−^, including intense diatropic regions both within and above the ring. These results clarify earlier interpretations—for instance, although Pham et al. [14] describe B_3_^+^ as π-aromatic only, the electronic transitions responsible for their reported ring currents involve E′-symmetric orbitals (HOMO–1,1′ → LUMO+1,1′), which are σ in nature, indicating previously overlooked σ-contributions. Finally, the stronger σ-delocalization in B_3_^−^ can be rationalized as arising from two cooperative delocalization pathways: one involving three 2c–2e σ-bonds and the other a radial 3c–2e σ-bond, the former structurally enabled by the availability of a third empty sp^2^ hybrid orbital on each boron atom for multicenter delocalization.

To complement the bonding analysis, we examined the Intrinsic Bond Orbitals (IBOs) of B_3_^−^ and B_3_^+^. In B_3_^−^, the IBOs recover three tangential σ-bonds with predominantly 2c–2e character, along with an in-plane delocalized orbital consistent with a radial 3c–2e σ-bond. A delocalized π-type orbital is also observed, in agreement with the AdNDP and EDDB results (Figure S4). By comparison, the IBOs of B_3_^+^ yield three σ-bonds more localized between adjacent atoms, though their spatial distribution suggests partial three-center character, and a delocalized π-orbital (Figure S5). The comparison reveals that σ-delocalization in B_3_^−^ involves both radial and tangential components acting cooperatively, whereas in B_3_^+^ it is restricted to a compact tangential framework. The IBO results thus provide a complementary picture that reinforces the distinct nature of σ-delocalization in each species and further supports the double aromatic character of B_3_^−^.

### 3.2. B_4_ and B_4_^2−^

The neutral B_4_ cluster, with a rhomboidal geometry and *D*_2h_ symmetry (Figure 1), has attracted considerable theoretical interest due to its electronic stability and non-trivial bonding. Initially characterized by Martin et al. and more thoroughly studied by Zubarev and Boldyrev [10], as well as Zhao et al. [56], it was established that its global minimum corresponds to the singlet ^1^A_g_ state. This results from a second-order Jahn–Teller distortion of a square geometry, with a shallow energy barrier (0.7–0.8 kcal/mol), supporting the idea that the system adopts a nearly square-like structure at ambient temperature.

Molecular orbital analyses identified a π-type HOMO–1 (1b_3u_) derived from 2p_z_ overlap and a σ-type HOMO (2a_g_) with a radial topology, analogous to that of B_3_^−^. Our AdNDP analysis (Figure S6) recovers one 4c–2e π-bond and one 4c–2e radial σ-bond, both with occupation numbers of 2.00 |e|. In addition, four 2c–2e σ-bonds are identified along the edges of the rhombus. Although AdNDP recovers these as localized, they can be seen as alternating with empty sp^2^ hybrid orbitals, forming a tangential 8-electron set that satisfies a 4n count (n = 2), analogous to the delocalization hypothesis proposed for B_3_^+^. Thus, the σ-electron framework in B_4_ can be formally decomposed into two sets of delocalized molecular orbitals: a radial set accommodating 2|e| (consistent with Hückel aromaticity, n = 0), and a tangential set accommodating 8|e| (formally 4n, with n = 2, indicative of antiaromatic character).

EDDB analysis (Figure S7) supports the presence of dual delocalization, yielding 3.4 |e| of total delocalized electrons—1.5|e| associated with the π-framework and 1.9 |e| with the σ-framework—indicating a modest predominance of σ-contributions. However, the MICD maps (Figure S8) reveal conflicting magnetic behavior: a diatropic π current of 3.9 nA·T^−1^ and a paratropic σ current of −5.1 nA·T^−1^, resulting in a net paratropic response of −1.2 nA·T^−1^. This interplay of opposing contributions agrees with GIMIC results reported by Zhao et al. [56] who described a diatropic inner current and an outer paratropic circulation. A 3.0 ppm isosurface $B_{z}^{ind}$ (Figure S9) displays a deshielded (paratropic) central region, corresponding to the σ-framework, surrounded by a shielded (diatropic) peripheral region associated with the π-electron delocalization. This magnetic description is more accurate than prior NICS calculations, where NICS(0) = −35.6 ppm, but the values at 0.5 and 1.0 Å (−24.5 and 7.7 ppm, respectively) reflect a shift toward paratropic behavior with increasing distance from the center.

In summary, B_4_ exhibits partial or conflicting aromaticity. The π-framework conforms to the 4n + 2 Hückel rule and displays consistent electronic and magnetic criteria of aromaticity. In contrast, the σ-framework, although electronically delocalized according to AdNDP and EDDB, yields a globally paratropic (antiaromatic) magnetic response. This apparent contradiction can be reconciled by dividing the σ-electrons into two sets: a radial 2-electron component (aromatic) and a tangential 8-electron component (antiaromatic). This interpretation aligns with the framework proposed for B_3_^−^ and B_3_^+^, where alternating occupied and vacant sp^2^ hybrid orbitals enable distinct delocalization pathways that account for their divergent magnetic behaviors.

On the other hand, the B_4_^2−^ cluster has been described as a square-planar species with *D*_4h_ symmetry and a singlet ^1^A_1g_ ground state. Sundholm et al. [27] first characterized it as an isoelectronic analogue of Al_4_^2−^. They proposed double (σ and π) aromaticity based on both bonding topology and magnetic response, reporting a ring current susceptibility (ARCS) of 7.6 nA·T^−1^. Zubarev and Boldyrev supported this view [10], identifying three delocalized 4c–2e orbitals: one π-type and two σ-type (radial and tangential). Our AdNDP analysis (Figure S10) offers a reinterpretation of the bonding topology, in which both localized 2c–2e and delocalized 4c–2e σ-bonds are jointly considered as part of a ten-electron tangential σ-framework. In addition, two delocalized 4c–2e bonds (one π and one σ-radial) are recovered. This electronic configuration satisfies the 4n + 2 Hückel rule for the π and radial, tangential σ-frameworks.

Delocalization is further supported by EDDB analysis (Figure S11), which reveals a total of 2.8 |e| delocalized: 1.5 |e| in the π-channel and 1.3 |e| in the σ-channel. This reflects a slightly stronger contribution from the π-framework and reverses the trend seen in neutral B_4_, where σ-delocalization dominated. From the magnetic viewpoint, the MICD maps (Figure S12) show strong diatropic currents in both frameworks. The integrated current strengths are 3.8 nA·T^−1^ (π) and 17.8 nA·T^−1^ (σ), yielding a total of 21.6 nA·T^−1^—fully consistent with cooperative double aromaticity and significantly stronger than the conflicting behavior in neutral B_4_. Complementarily, the $B_{z}^{\mathrm{ind}}$ isosurface at 3.0 ppm (Figure S13), displaying a uniform diatropic shielding topology, with blue-shaded (shielded) regions throughout and no paratropic contributions. This contrasts with B_4_, where the tangential σ-electrons formed a 4n system associated with paratropic (antiaromatic) response.

To further validate the bonding patterns, we performed Intrinsic Bond Orbital (IBO) analyses for B_4_ and B_4_^2−^ at the same level of theory. For B_4_ (Figure S14), the IBOs reproduce the bonding framework derived from AdNDP: four 2c–2e σ-bonds localized along the rhombus edges, one 4c–2e σ-orbital with radial character, and one 4c–2e π-orbital. This excellent agreement confirms the robustness of the AdNDP picture and supports the interpretation of a σ-aromatic component through radial delocalization. In the case of B_4_^2−^ (Figure S15), IBO also identifies four 2c–2e σ-bonds at the edges, as well as a 4c–2e π-orbital. However, instead of distinct radial and tangential σ-delocalized orbitals as in AdNDP, the IBO analysis yields two 4c–2e σ-orbitals that appear to mix both components. This difference arises from the inherently localized and orthonormal nature of IBOs, which may combine overlapping delocalization pathways. Nonetheless, these orbitals can still be related to the AdNDP-derived radial and tangential motifs, and their presence supports the overall picture of dual σ- and π-delocalization. The convergence between both methods reinforces the interpretation of B_4_^2−^ as a doubly aromatic system.

### 3.3. The Case of B_4_^2+^

**Figure 3:**
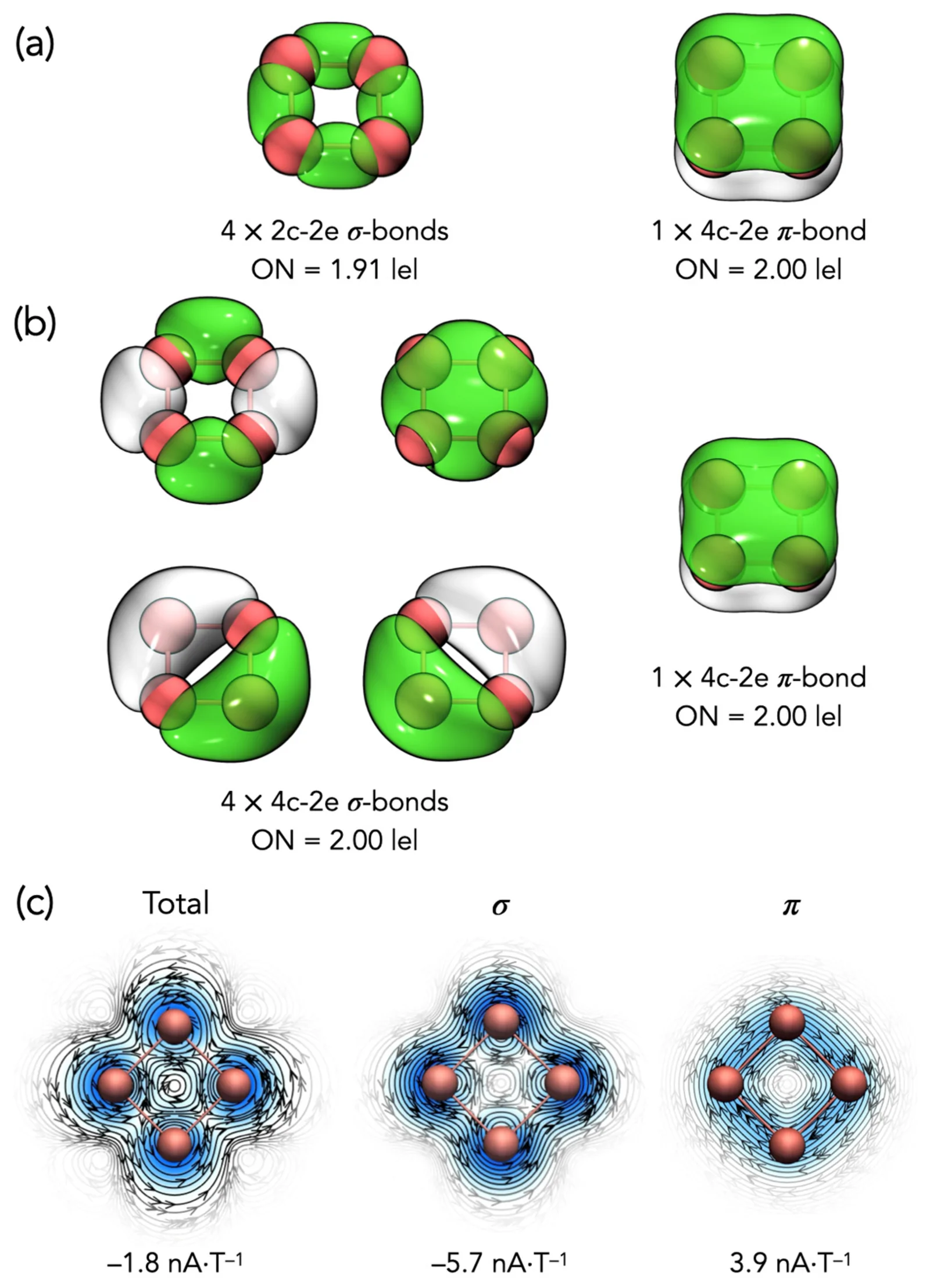
AdNDP and MICD analysis of B_4_^2+^. (**a**) Localized AdNDP solution (isovalue = ±0.05). (**b**) Delocalized AdNDP solution (isovalue = ±0.05). (**c**) Total, σ- and π-MICD maps 0.5 Å above the molecular plane, with corresponding RCS values (nA·T^−1^), revealing σ-paratropic and π-diatropic responses.

To further examine the correlation between electronic structure and magnetic response, we analyzed the B_4_^2−^ dication. The AdNDP bonding pattern shown in Figure 3a reveals four localized 2c–2e σ-bonds and one delocalized 4c–2e π-bond, accounting for eight tangential σ-electrons and two π-electrons. The MICD maps in Figure 3c display a pronounced paratropic σ-ring current (−5.7 nA·T^−1^) and a diatropic π-ring current (3.9 nA·T^−1^), resulting in a net paratropic response (−1.8 nA·T^−1^). These features are fully consistent with Hückel’s 4n rule for the σ-system and 4n + 2 for the π-system, supporting a description of σ-antiaromaticity and π-aromaticity.

Alternatively, we guided the AdNDP search toward delocalized bonding (Figure 3b). This yielded three delocalized 4c–2e σ-orbitals of tangential character, along with one 4c–2e σ-orbital of radial character and one 4c–2e π-orbital. This alternative representation reinforces the partitioning scheme proposed for B_3_^−^, B_3_^+^, B_4_, and B_4_^2−^ in which tangential σ-electrons—although often recovered as localized—alternate with vacant hybrid orbitals and can be effectively treated as a delocalized 4n system. In B_4_^2+^, this partition provides a rationale for the observed paratropic σ-response and confirms the utility of grouping tangential σ-electrons when applying Hückel’s rule in interpreting MICD results.

The IBO analysis (Figure S16) retrieves the same bonding pattern as the localized AdNDP representation—four 2c–2e σ-bonds and one 4c–2e π-bond—thus preserving the same electron counts within each framework. This result further supports the interpretation of a tangential 4n σ-system and a π-framework consistent with the 4n + 2 rule.

### 3.4. Additional Arguments to Support Two Circuit σ-Delocalization

**Figure 4:**
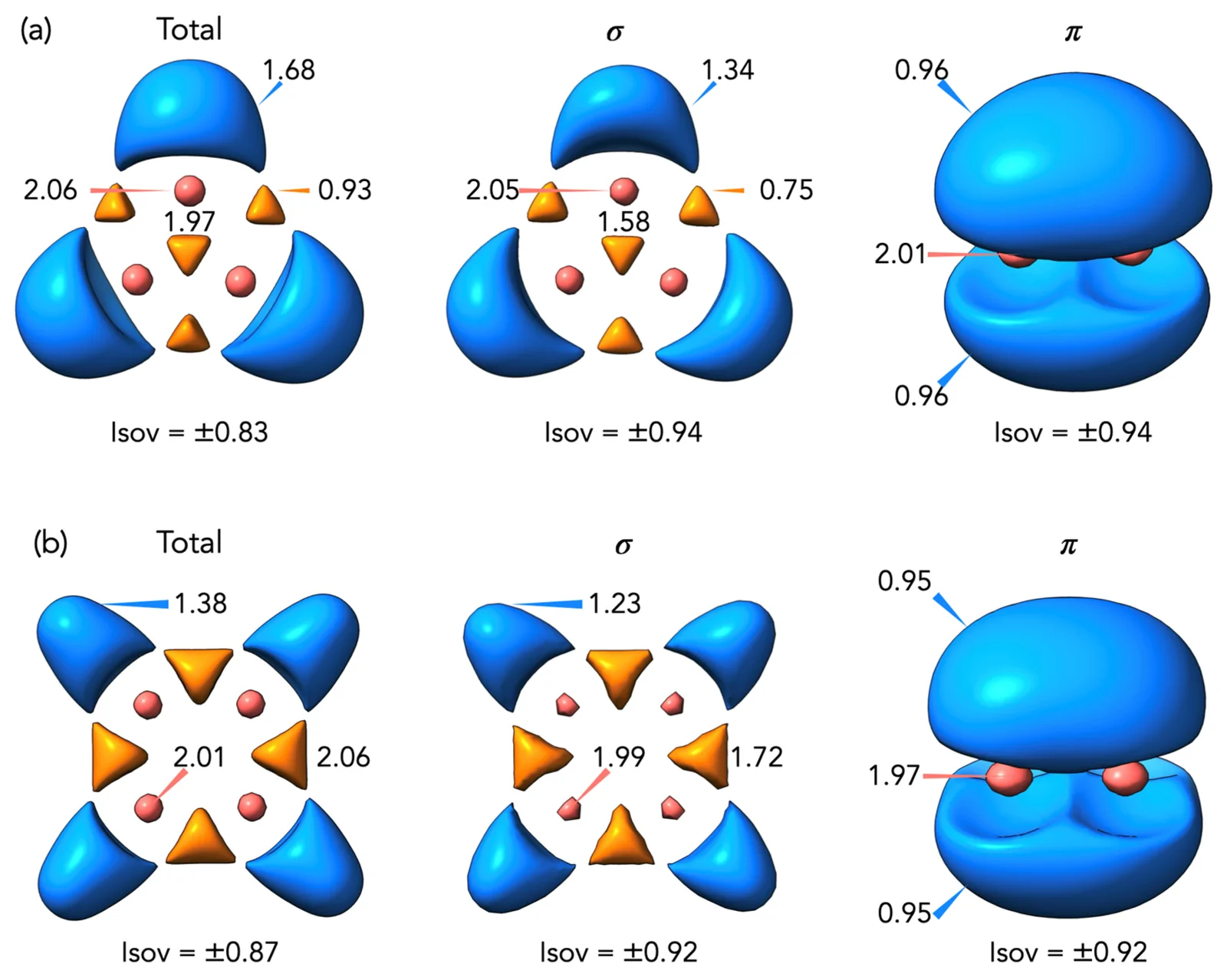
ELF isosurfaces for (**a**) B_3_^−^ and (**b**) B_4_^2−^, decomposed into total (**left**), σ (**center**), and π (**right**) contributions. Isovalues are indicated for each plot. Light blue lobes correspond to lone-pair basins, orange to 2c–2e B–B bond basins, and pink to core basins. The electron population of each basin is labeled.

To further support our bonding model and justify the inclusion of 2c–2e σ-bonds within extended delocalization circuits, we performed Electron Localization Function (ELF) [57,58] analyses for the B_3_^−^ and B_4_^2−^ clusters, shown in Figure 4. These three- and four-membered systems, with 10 and 14 valence electrons, respectively, serve as representative cases to probe electron distribution and bonding topology. The ELF isosurfaces—resolved into total, σ-, and π-contributions—reveal clear signatures of electron deficiency and multicenter bonding. Light blue basins, associated with lone-pair-like regions, display electron populations well below the expected 2.0 e for fully localized lone pairs, ranging from 1.68 |e| (total) to 1.23 |e| (σ-only), confirming that the σ-framework itself is intrinsically electron-deficient. Similarly, bonding basins (orange) show sub-2.0 |e| populations: in B_3_^−^, σ-bond basins exhibit 0.75 |e| in the σ-partitioned ELF and 0.93 |e| in the total ELF, while in B_4_^2−^ they range from 1.72 *e* (σ) to 2.06 |e| (total). Notably, in B_3_^−^, a multicenter σ-basin of 1.97 |e| is also recovered, consistent with delocalized three-center bonding.

These ELF results align with the view that σ-delocalization in these electron-deficient clusters involves not only conventional bonding regions but also partially populated lone-pair zones, interpreted here as sp^2^-like hybrid orbitals. Globally, the average electron count per boron atom is ~4.2 |e| in B_3_^−^ and ~5.5 |e| in B_4_^2−^—well below the octet—highlighting the need for multicenter electron sharing. Within this framework, the σ-delocalized circuit alternates between partially filled bond basins and lone-pair-depleted regions. The observed basin connectivity and population patterns provide strong evidence for including all σ-bonds topologically embedded in the tangential conjugation pathway as contributors to aromatic delocalization. This interpretation is fully consistent with the AdNDP and IBO analyses, as well as with the ring-current patterns observed in the MICD results.

### 3.5. Integrating AdNDP, EDDB, and MICD Analyses: Consistency Across the Series

Table S1 compiles, for all clusters, the σ/π EDDB delocalization values (*e*), the 4n or 4n + 2 electron counts from AdNDP (distinguishing σ_rad_, σ_tan_, and π), and the dissected MICD RCSs (σ_rad+tan_ and π, in nA·T^−1^). Clear trends emerge: B_3_^−^ and B_4_^2−^ satisfy 4n + 2 in both σ and π and sustain strong co-diatropic net ring currents (total RCS 16.0 and 21.6 nA·T^−1^, respectively). In contrast, B_4_ and B_4_^2+^ meet 4n + 2 only in π (σ_tan_ = 4n), yielding paratropic σ currents (−5.1 and −5.7 nA·T^−1^) and net antiaromatic responses (−1.2 and −1.8 nA·T^−1^). B_3_^+^ retains 4n + 2 in π and σ_tan_ and exhibits coexisting diatropic σ/π currents (total 11.2 nA·T^−1^; σ = 7.4, π = 3.8). Benchmarks behave as expected: C_6_H_6_ shows a dominant π-diatropic current (12.2 nA·T^−1^), whereas C_4_H_4_ is strongly antiaromatic (−21.0 nA·T^−1^). Overall, the table shows close agreement among AdNDP counts, EDDB delocalization, and MICD-derived ring currents across the series.

## 4. Conclusions

A unified framework for interpreting aromaticity in small boron rings (B_3_^−^, B_3_^+^, B_4_, B_4_^2−^, and B_4_^2+^) has been established by integrating AdNDP, IBO, EDDB, MICD, and $B_{z}^{ind}$ analyses. By separating the σ-framework into radial (σᵣ) and tangential (σₜ) components, Hückel’s rule can be applied independently to each circuit, revealing three possible σ-regimes: (i) doubly σ-aromatic (4n + 2 in both σᵣ and σₜ), (ii) σ-conflicting aromaticity (one σ-component 4n + 2, the other 4n), and (iii) σ-antiaromaticity (4n in both). In triangular species, σᵣ corresponds to a 3c–2e bond, whereas in rhomboidal species it arises from a 4c–2e bond; σₜ is described as alternating 2c–2e bonds and empty or partially filled sp^2−^ like sites, forming delocalized 4n or 4n + 2 sets.

Within this scheme, B_3_^−^ (σᵣ = 2 |e|, σₜ = 6 |e|, π = 2 |e|) and B_4_^2−^ (σᵣ = 2 |e|, σₜ = 10 |e|, π = 2 |e|) are doubly σ-aromatic and π-aromatic. B_3_^+^ lacks σᵣ but retains σₜ = 6 |e| and π = 2 |e|, leading to coexisting σₜ/π aromaticity. B_4_ (σᵣ = 2 |e|, σₜ = 8 |e|, π = 2 |e|) exhibits σ-conflicting aromaticity, where σᵣ is aromatic but σₜ is antiaromatic (4n), producing a net paratropic σ-response dominated by the σₜ circuit. In contrast, B_4_^2+^ (σₜ = 8 e, π = 2 |e|) shows net conflicting aromaticity, with σ antiaromaticity and π aromaticity. The consistency between bonding patterns, electron delocalization, and magnetic responses validates this classification and clarifies prior misassignments, such as the σₜ aromaticity of B_3_^+^ and the net aromatic character of B_4_.

## Figures and Tables

**Table 1:** NICS values previously reported for reexamine B_3_^−^, B_3_^+^, B_4_, and B_4_^2−^, along with reference systems C_6_H_6_ and C_4_H_4_, at various distances above the ring center, compared with total ring current strengths (RCS) and σ/π separation calculated in this work. The “Agreement” column indicates the consistency between magnetic aromaticity measures (NICS and RCS) and the 4n/4n + 2 electron counts obtained from canonical molecular orbital (CMO) analyses reported in prior studies.

Scheme	NICS	CMO		RCS			Agreement
		σ	π	σ	π	Total	
B_3_^−^	−73.6 [10] (0.0 Å) −57.9 [10] (0.5 Å) −28.2 [10] (1.0 Å)	4n + 2 [10,14]	4n + 2 [10,14]	12.1	3.9	16.0	**✓**
B_3_^+^	−66.3 [10] (0.0 Å) −46.3 [10] (0.5 Å) −15.9 [10] (1.0 Å)	__	4n + 2 [10,14]	7.4	3.8	11.2	**✕**
B_4_	−35.6 [10](0.0 Å) −24.5 [10] (0.5 Å) 7.7 [10] (1.0 Å)	4n + 2 [10]	4n + 2 [10,14]	−5.1	3.9	−1.2	**✕**
B_4_^2−^	−29.5 [53] (0.0 Å) −3.0 [53] (1.25 Å)	4n + 2 [14,27]	4n + 2 [14,27]	17.8	3.8	21.6	**✓**
C_6_H_6_	−8.2 [26,54] (0.0 Å) −9.8 [26,54] (0.5 Å) −10.2 [26,54] (1.0 Å)	__	4n + 2 [26,54]	0.3	11.9	12.2	**✓**
C_4_H_4_	+21.5 [26,54] (0.0 Å) +13.3 [26,54] (1.0 Å)	__	4n [26,54]	−5.4	−15.6	−21.0	**✓**

## Data Availability

The original contributions presented in this study are included in the article/Supplementary Material. Further inquiries can be directed to the corresponding authors.

## Supplementary Materials

The following supporting information can be downloaded at: at https://www.mdpi.com/article/10.3390/molecules30173566/s1, Figures S1, S3, S9 and S13: Out-of-plane component of the induced magnetic field ($B_{z}^{ind}$), shown as an isosurface (left, isovalue = −3.00 ppm) and 2D contour maps on perpendicular planes of B_3_^−^, B_3_^+^, B_4_, and B_4_^2−^, respectively. Figures S2, S6 and S10: AdNDP bonding pattern of the B_3_^+^, B_4_ and B_4_^2^, respectively. Figures S4, S5, S14–S16: Intrinsic Bond Orbitals (IBOs) of B_3_^−^, B_3_^+^, B_4_, B_4_^2−^, and B_4_^2+^. Figures S7 and S11: EDDB_G_ isosurfaces of B_4_ and B_4_^2−^, respectively. Figures S8 and S12: Total, σ- and π-MICD maps 0.5 Å above the molecular plane, with corresponding RCS values of B_4_ and B_4_^2−^, respectively. Table S1: Integrating AdNDP, EDDB, and MICD Analyses of the B_3_^−^, B_3_^+^, B_4_, B_4_^2+^, B_4_^2−^, C_6_H_6_ and C_4_H_4_. Table S2: Cartesian coordinates of the B_3_^−^, B_3_^+^, B_4_, B_4_^2+^, and B_4_^2−^ optimized structures at the PBE0-D3/def2-TZVP level.

## References

[1] von Schleyer P.R., Jiao H. (1996). What Is Aromaticity?. Pure Appl. Chem..

[2] von Ragué Schleyer P. (2001). Introduction: Aromaticity. Chem. Rev..

[3] Krygowski T.M., Cyrański M.K. (2001). Structural Aspects of Aromaticity. Chem. Rev..

[4] Fernández I. (2021). Aromaticity: Modern Computational Methods and Applications.

[5] Zhai H., Averkiev B.B., Zubarev D.Y., Wang L., Boldyrev A.I. (2007). δ Aromaticity in [Ta_3_O_3_]^−^. Angew. Chemie..

[6] Zubarev D.Y., Averkiev B.B., Zhai H.-J., Wang L.-S., Boldyrev A.I. (2008). Aromaticity and Antiaromaticity in Transition-Metal Systems. Phys. Chem. Chem. Phys..

[7] Boldyrev A.I., Wang L.-S. (2005). All-Metal Aromaticity and Antiaromaticity. Chem. Rev..

[8] Zubarev D.Y., Boldyrev A.I. (2008). Developing Paradigms of Chemical Bonding: Adaptive Natural Density Partitioning. Phys. Chem. Chem. Phys..

[9] Rincon L., Almeida R., Alvarellos E., Garcia-aldea D., Hasmy A., Gonzales C. (2009). The Role Delocalization in Planar Boron Clusters. Dalt. Trans..

[10] Zubarev D.Y.U., Boldyrev A.I. (2006). Comprehensive Analysis of Chemical Bonding in Boron Clusters. J. Comput. Chem..

[11] Alexandrova A.N., Boldyrev A.I., Zhai H.-J., Wang L.-S. (2006). All-Boron Aromatic Clusters as Potential New Inorganic Ligands and Building Blocks in Chemistry. Coord. Chem. Rev..

[12] Aihara J., Kanno H., Ishida T. (2005). Aromaticity of Planar Boron Clusters Confirmed. J. Am. Chem. Soc..

[13] Barroso J., Pan S., Merino G. (2022). Structural Transformations in Boron Clusters Induced by Metal Doping. Chem. Soc. Rev..

[14] Pham H.T., Lim K.Z., Havenith R.W.A., Nguyen M.T. (2016). Aromatic Character of Planar Boron-Based Clusters Revisited by Ring Current Calculations. Phys. Chem. Chem. Phys.

[15] Tsipis C.A. (2010). Aromaticity/Antiaromaticity in “Bare” and “Ligand-Stabilized” Rings of Metal Atoms. Metal-Metal Bonding.

[16] Peerless B., Schmidt A., Franzke Y.J., Dehnen S. (2023). φ-Aromaticity in Prismatic {Bi_6_}−Based Clusters. Nat. Chem..

[17] Dari C., Leyva-Parra L., Yang Y., Tiznado W., Cui Z. (2025). Ga_5_Li_12_^+^: A Doubly Aromatic Ga_5_^11–^ Ring Stabilized by Lithium Cations. Inorg. Chem..

[18] Feixas F., Matito E., Poater J., Solà M. (2015). Quantifying Aromaticity with Electron Delocalisation Measures. Chem. Soc. Rev..

[19] Báez-Grez R., Ruiz L., Pino-Rios R., Tiznado W. (2018). Which NICS Method Is Most Consistent with Ring Current Analysis? Assessment in Simple Monocycles. RSC Adv..

[20] Inostroza D., García V., Yañez O., Torres-Vega J.J., Vásquez-Espinal A., Pino-Rios R., Báez-Grez R., Tiznado W. (2021). On the NICS Limitations to Predict Local and Global Current Pathways in Polycyclic Systems. New J. Chem..

[21] Solà M. (2022). Aromaticity Rules. Nat. Chem..

[22] Torres-Vega J.J., Vásquez-Espinal A., Caballero J., Valenzuela M.L., Alvarez-Thon L., Osorio E., Tiznado W. (2014). Minimizing the Risk of Reporting False Aromaticity and Antiaromaticity in Inorganic Heterocycles Following Magnetic Criteria. Inorg. Chem..

[23] Schleyer P.V.R., Pühlhofer F. (2002). Recommendations for the Evaluation of Aromatic Stabilization Energies. Org. Lett..

[24] George P., Trachtman M., Bock C.W., Brett A.M. (1976). Homodesmotic Reactions for the Assessment of Stabilization Energies in Benzenoid and Other Conjugated Cyclic Hydrocarbons. J. Chem. Soc. Perkin Trans. 2.

[25] Mo Y., Song L., Lin Y. (2007). Block-Localized Wavefunction (BLW) Method at the Density Functional Theory (DFT) Level. J. Phys. Chem. A.

[26] Chen Z., Wannere C.S., Corminboeuf C., Puchta R., Schleyer P.V.R. (2005). Nucleus-Independent Chemical Shifts (NICS) as an Aromaticity Criterion. Chem. Rev..

[27] Jusélius J., Straka M., Sundholm D. (2001). Magnetic-Shielding Calculations on Al_4_^2−^ and Analogues. A New Family of Aromatic Molecules?. J. Phys. Chem. A.

[28] Geuenich D., Hess K., Köhler F., Herges R. (2005). Anisotropy of the Induced Current Density (ACID), a General Method To Quantify and Visualize Electronic Delocalization. Chem. Rev..

[29] Sundholm D., Berger R.J.F., Fliegl H. (2016). Analysis of the Magnetically Induced Current Density of Molecules Consisting of Annelated Aromatic and Antiaromatic Hydrocarbon Rings. Phys. Chem. Chem. Phys..

[30] Sundholm D., Fliegl H., Berger R.J.F. (2016). Calculations of Magnetically Induced Current Densities: Theory and Applications. WIREs Comput. Mol. Sci..

[31] Leyva-Parra L., Pino-Rios R., Inostroza D., Solà M., Alonso M., Tiznado W. (2024). Aromaticity and Magnetic Behavior in Benzenoids: Unraveling Ring Current Combinations. Chem.–A Eur. J..

[32] Liu Z., Lu T., Chen Q. (2020). An Sp-Hybridized All-Carboatomic Ring, Cyclo[18]Carbon: Bonding Character, Electron Delocalization, and Aromaticity. Carbon N. Y..

[33] Kruszewski J., Krygowski T.M. (1972). Definition of Aromaticity Basing on the Harmonic Oscillator Model. Tetrahedron Lett..

[34] Szczepanik D.W., Żak E., Dyduch K., Mrozek J. (2014). Electron Delocalization Index Based on Bond Order Orbitals. Chem. Phys. Lett..

[35] Ghosh S.R., Halder S.C., Mitra S., Mondal R., Jana A.D. (2025). Evolution of Aromaticity in B_3_^n^ (n = 2+, 1+, 0, 1−, 2−, 3−) Clusters upon Electron Injection and Abstraction: A Comprehensive Analysis Using ELF, NICS, Ring Current Maps and AdNDP. Comput. Theor. Chem..

[36] Tai T.B., Ceulemans A., Nguyen M.T. (2012). Disk Aromaticity of the Planar and Fluxional Anionic Boron Clusters B_20_^−^/^2−^. Chem.—A Eur. J..

[37] Dordević S., Solà M., Radenković S. (2022). Aromaticity of Singlet and Triplet Boron Disk-like Clusters: A Test for Electron Counting Aromaticity Rules. Inorg. Chem..

[38] Li X., Kuznetsov A.E., Zhang H.-F., Boldyrev A.I., Wang L.-S. (2001). Observation of All-Metal Aromatic Molecules. Science.

[39] Adamo C., Barone V. (1999). Toward Reliable Density Functional Methods without Adjustable Parameters: The PBE0 Model. J. Chem. Phys..

[40] Grimme S., Antony J., Ehrlich S., Krieg H. (2010). A Consistent and Accurate Ab Initio Parametrization of Density Functional Dispersion Correction (DFT-D) for the 94 Elements H-Pu. J. Chem. Phys..

[41] Weigend F., Ahlrichs R. (2005). Balanced Basis Sets of Split Valence, Triple Zeta Valence and Quadruple Zeta Valence Quality for H to Rn: Design and Assessment of Accuracy. Phys. Chem. Chem. Phys..

[42] Frisch M.J., Trucks G.W., Schlegel H.B., Scuseria G.E., Robb M.A., Cheeseman J.R., Scalmani G., Barone V., Petersson G.A., Nakatsuji H. (2016). Gaussian 16.

[43] Lu T., Chen F. (2012). Multiwfn: A Multifunctional Wavefunction Analyzer. J. Comput. Chem..

[44] Humphrey W., Dalke A., Schulten K. (1996). VMD: Visual molecular dynamics. J. Mol. Graph..

[45] Knizia G., Klein J.E.M.N. (2015). Electron Flow in Reaction Mechanisms—Revealed from First Principles. Angew. Chem. Int. Ed..

[46] Knizia G. (2013). Intrinsic Atomic Orbitals: An Unbiased Bridge between Quantum Theory and Chemical Concepts. J. Chem. Theory Comput..

[47] Neese F. (2022). Actualización de Software: El Sistema Del Programa ORCA—Versión 5.0. WIREs Comput. Mol. Sci..

[48] Gerald Knizia Group (2013). IboView.

[49] Monaco G., Summa F.F., Zanasi R. (2021). Program Package for the Calculation of Origin-Independent Electron Current Density and Derived Magnetic Properties in Molecular Systems. J. Chem. Inf. Model..

[50] Cheeseman J.R., Trucks G.W., Keith T.A., Frisch M.J. (1996). A Comparison of Models for Calculating Nuclear Magnetic Resonance Shielding Tensors. J. Chem. Phys..

[51] Lehtola S., Dimitrova M., Fliegl H., Sundholm D. (2021). Benchmarking Magnetizabilities with Recent Density Functionals. J. Chem. Theory Comput..

[52] Zhai H., Wang L., Alexandrova A.N., Boldyrev A.I., Zakrzewski V.G. (2003). Photoelectron Spectroscopy and Ab Initio Study of B_3_^−^ and B_4_^−^ Anions and Their Neutrals. J. Phys. Chem. A.

[53] Nigam S., Majumder C., Kulshreshtha S.K. (2006). Theoretical Study of Aromaticity in Inorganic Tetramer Clusters. J. Chem. Sci..

[54] Corminboeuf C., Heine T., Weber J. (2003). Evaluation of Aromaticity: A New Dissected NICS Model Based on Canonical Orbitals. Phys. Chem. Chem. Phys..

[55] Ranjan S., Charan S., Mitra S., Mondal R., Dipankar A. (2025). Journal of Molecular Graphics and Modelling Unravelling the Effect of Successive Electron Injection into the Smallest Cyclic Boron Cluster, B_n_ Structure Analysis. J. Mol. Graph. Model..

[56] Zhao D., He X., Li M., Wang B., Guo C., Rong C., Chattaraj P.K., Liu S. (2021). Density Functional Theory Studies of Boron Clusters with Exotic Properties in Bonding, Aromaticity and Reactivity. Phys. Chem. Chem. Phys..

[57] Becke A.D., Edgecombe K.E. (1990). A Simple Measure of Electron Localization in Atomic and Molecular Systems. J. Chem. Phys..

[58] Tian L., Chen F.W. (2011). Meaning and Functional Form of the Electron Localization Function. Acta Phys.-Chim. Sin..

