# Supplementary material for: Interpreting Ring Currents from Hückel-Guided σ- and π-Electron Delocalization in Small Boron Rings
Source: Molecules. 2025 Aug 31;30(17):3566. doi: 10.3390/molecules30173566 (PMC12430551; doi:10.3390/molecules30173566)
Supplement: Supplementary file 1 [file molecules-30-03566-s001.zip › molecules-3792637-supplementary.pdf]

SUPPORTING INFORMATION

Interpreting Ring Currents from Hückel-Guided σ- and π-Electron Delocalization in Small Boron Rings

Dumer S. Sacanamboy,[1,2] Williams García-Argote,[2] Rodolfo Pumachagua-Huertas,[3] Carlos Cárdenas,[4,5] Luis Leyva-Parra,[2] Lina Ruiz[6], * and William Tiznado[2,*]

1. Doctorado en Fisicoquímica Molecular, Facultad de Ciencias Exactas, Universidad Andrés Bello, República 275, Santiago 837014, Chile.
2. Centro de Investigación para el Diseño de Materiales (CEDEM), Facultad de Ciencias Exactas, Departamento de Ciencias Químicas, Universidad Andrés Bello, Avenida República 275, Santiago 837014, Chile.
3. Laboratorio de Investigación en Química Teórica, Escuela Profesional de Química, Facultad de Ciencias Naturales y Matemáticas, Universidad Nacional Federico Villarreal, Jr. Río Chepén 290, El Agustino, Lima, Perú.
4. Departamento de Física, Facultad de Ciencias, Universidad de Chile, Av. Las Palmeras 3425, Ñuñoa, Santiago, Chile
5. Center for Delevopment of Nanoscience and Nanotechnology (CEDENNA), Av. Libertador Bernardo O Higgins 3363, Santiago, Chile
6. Institute of Biomedical Sciences, Faculty of Health Sciences, Universidad Autónoma de Chile, Santiago 8910060, Chile

*Correspondence: lina.ruiz@uautonoma.cl (L.R.); wtiznado@unab.cl (W.T.).

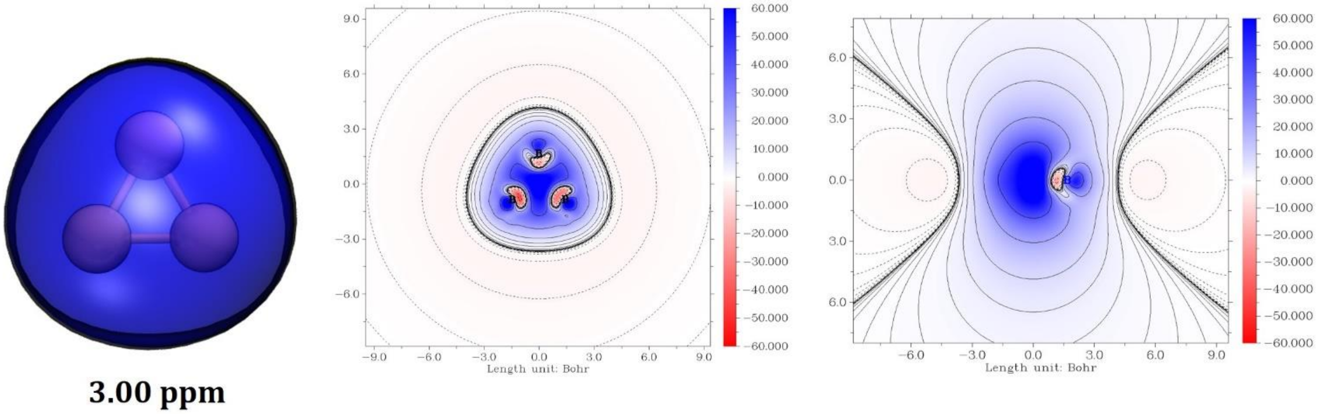

Figure S1. Out-of-plane component of the induced magnetic field (B_z^{ind}), shown as an isosurface (left, isovalue = –3.00 ppm) and 2D contour maps on perpendicular planes of the B_3^- system (center and right). Calculated at the BHandHLYP/def2-TZVP//PBE0-D3/def2-TZVP level of theory. Blue surfaces correspond to regions of magnetic shielding, while red surfaces indicate deshielding.

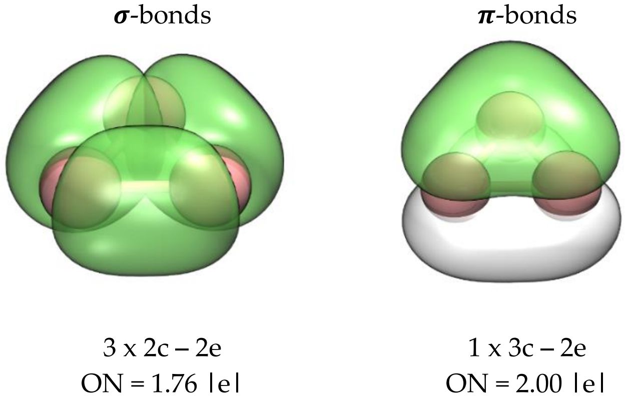

Figure S2. Adaptive Natural Density Partitioning (AdNDP) bonding pattern of the B_3^+ system computed at the PBE0-D3/def2-TZVP level of theory. Occupation numbers (ON) are given in |e|(isovalue = ±0.05).

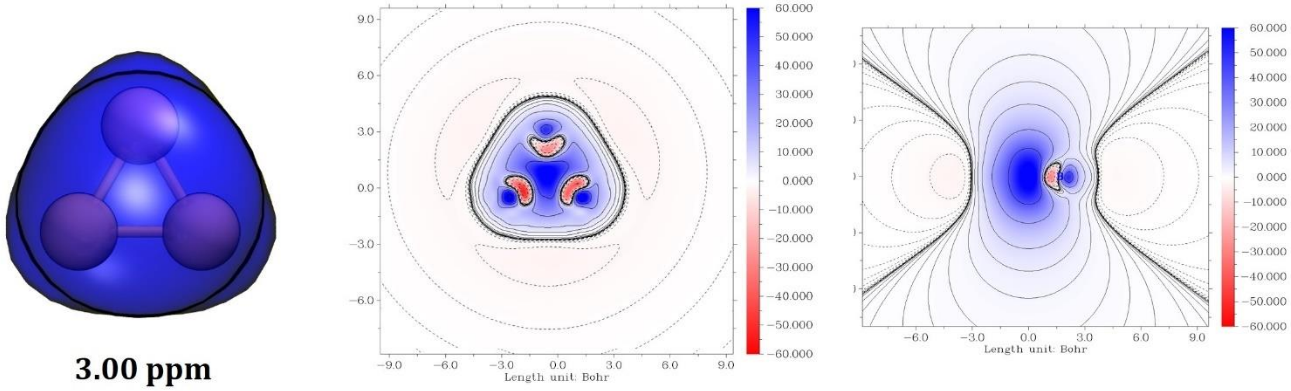

Figure S3. Out-of-plane component of the induced magnetic field (B_z^{ind}), shown as an isosurface (left, isovalue = –3.00 ppm) and 2D contour maps on perpendicular planes of the B_3^+ system (center and right). Calculated at the BHandHLYP/def2-TZVP//PBE0-D3/def2-TZVP level of theory. Blue surfaces correspond to regions of magnetic shielding, while red surfaces indicate deshielding.

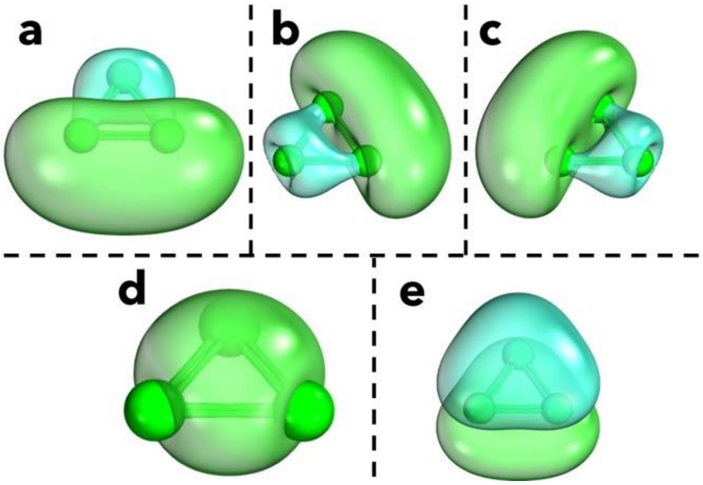

Figure S4. Intrinsic Bond Orbitals (IBOs) of B_3^-: (a–c) three tangential B–B σ bonds (2c–2e), (d) one radial σ bond (3c–2e), and (e) one π bond (3c–2e) delocalized over the three atoms.

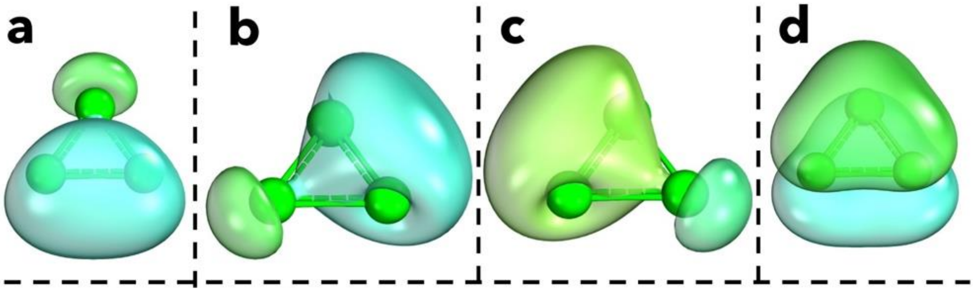

Figure S5. Intrinsic Bond Orbitals (IBOs) of B_3^+: (a–c) three B–B σ bonds with tangential orientation (2c–2e) and (d) one delocalized π bond (3c–2e).

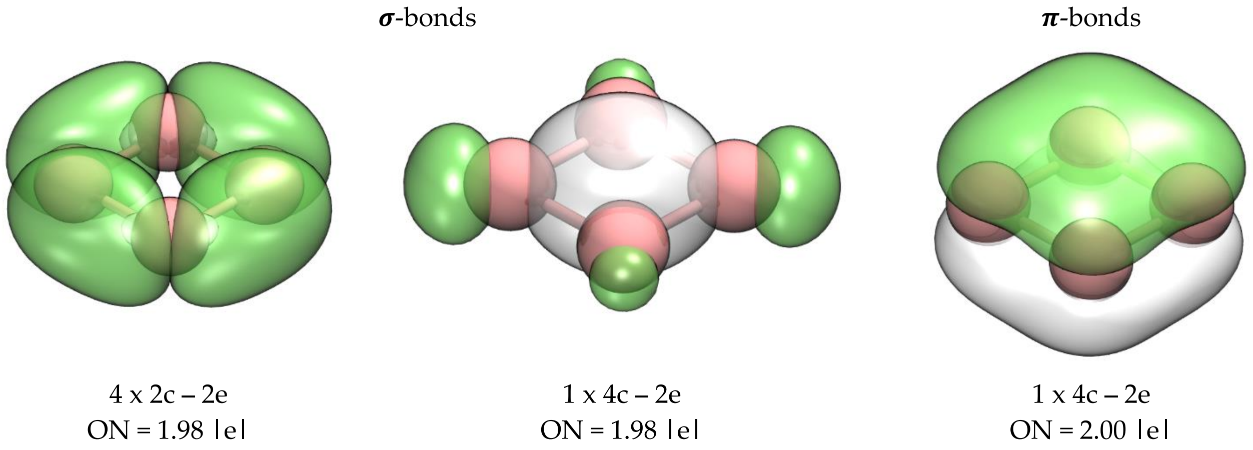

Figure S6. Adaptive Natural Density Partitioning (AdNDP) bonding pattern of the B_4 system computed at the PBE0-D3/def2-TZVP level of theory. Occupation numbers (ON) are given in |e| (isovalue = ±0.05).

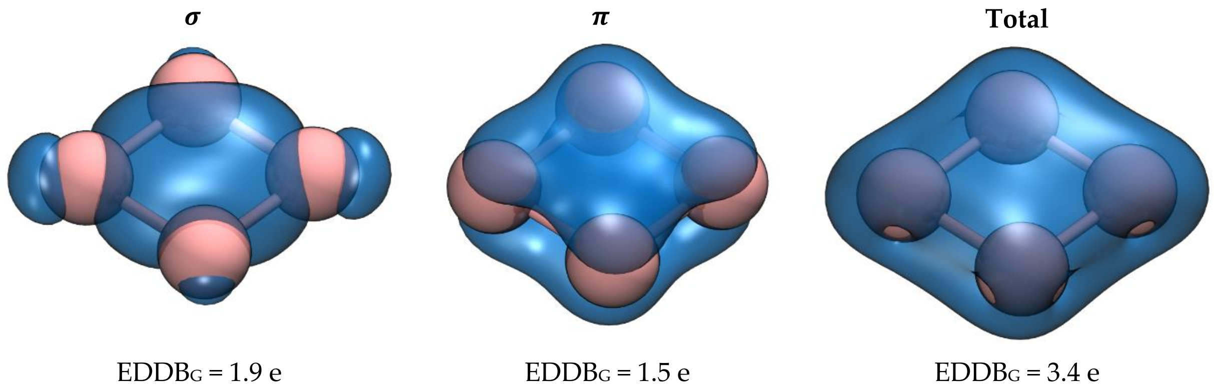

Figure S7. The electron density of delocalized bonds (EDDB) isosurfaces of B_4 (PBE0-D3/def2-TZVP) showing total, σ, and π delocalization. Electron populations in |e| (isovalue = ±0.001).

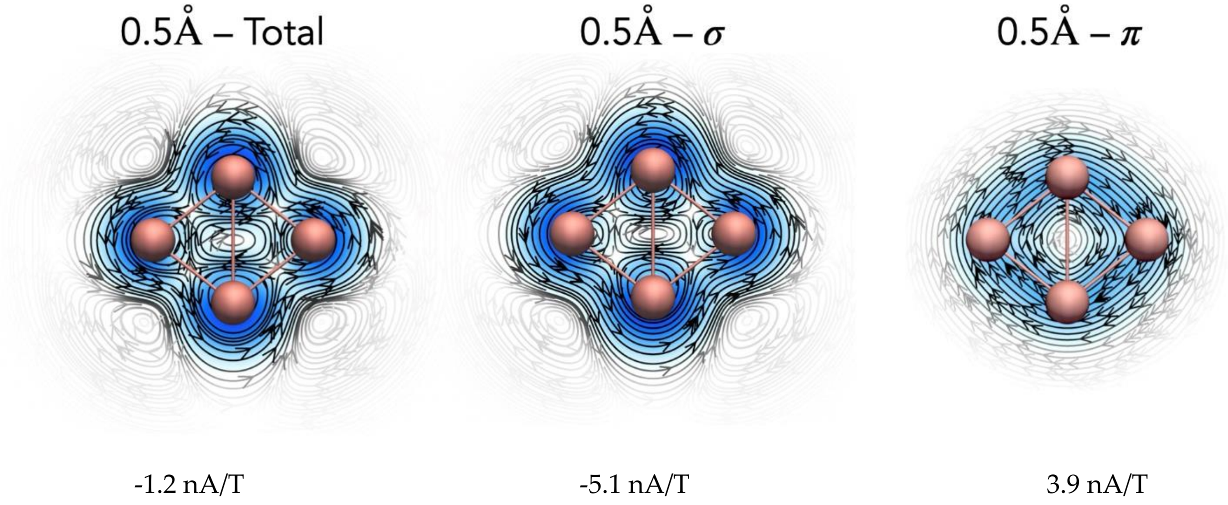

Figure S8. Total, σ- and π-MICD maps 0.5 Å above the molecular plane, with corresponding RCS values of B_4. Calculated at the BHandHLYP/def2-TZVP//PBE0-D3/def2-TZVP level.

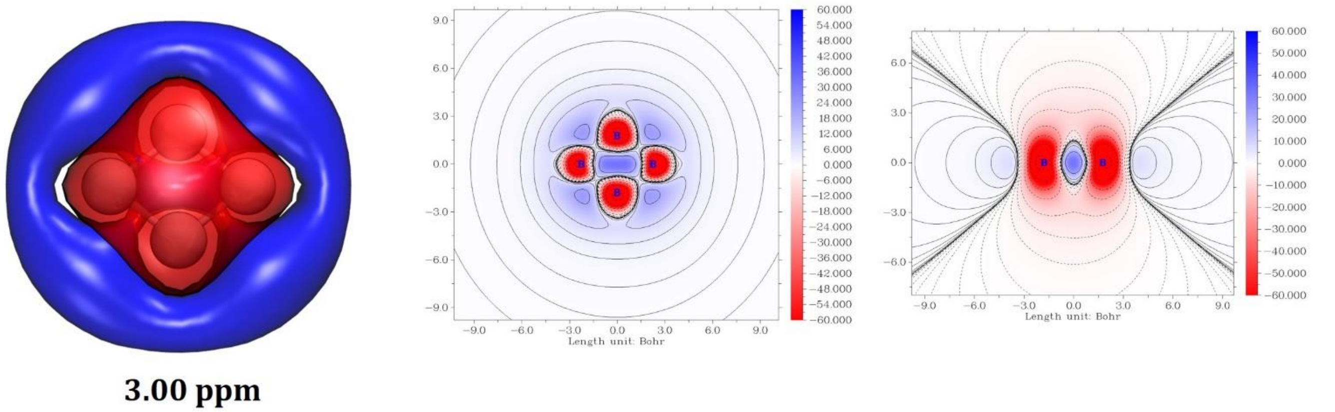

Figure S9. Out-of-plane component of the induced magnetic field (B_z^{ind}), shown as an isosurface (left, isovalue = –3.00 ppm) and 2D contour maps on perpendicular planes of the B_4 system (center and right). Calculated at the BHandHLYP/def2-TZVP//PBE0-D3/def2-TZVP level of theory. Blue surfaces correspond to regions of magnetic shielding, while red surfaces indicate deshielding.

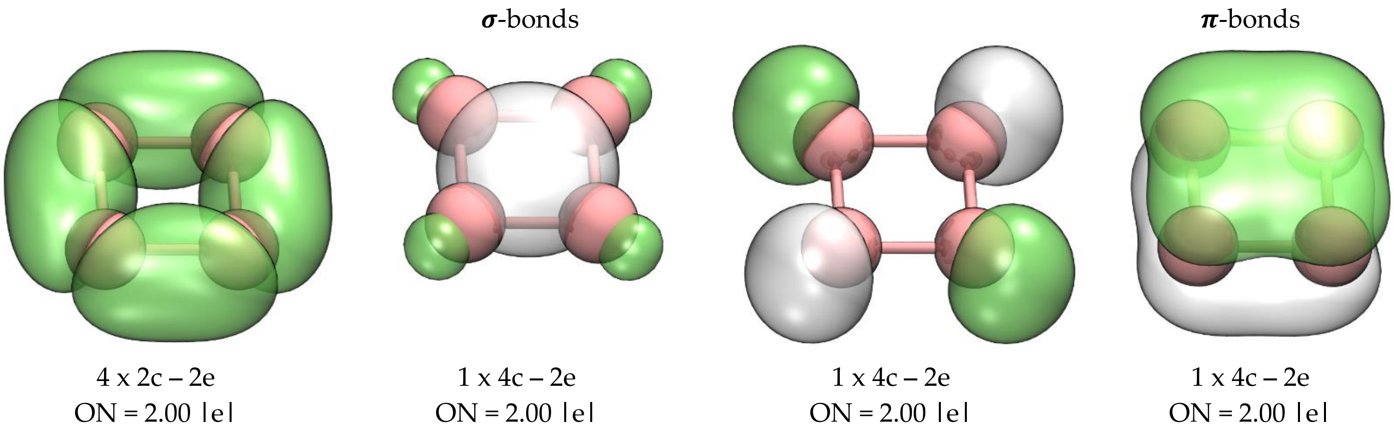

Figure S10. Adaptive Natural Density Partitioning (AdNDP) bonding pattern of the B_4^{2-} system computed at the PBE0-D3/def2-TZVP level of theory. Occupation numbers (ON) are given in |e|(isovalue = ±0.05).

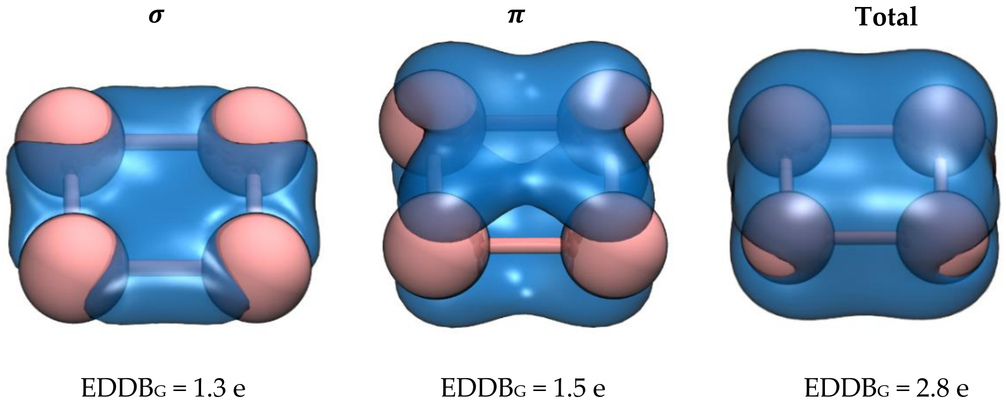

Figure S11. The electron density of delocalized bonds (EDDB) isosurfaces of B_4^{2-} (PBE0-D3/def2-TZVP) showing total, σ, and π delocalization. Electron populations in |e| (isovalue = ±0.001).

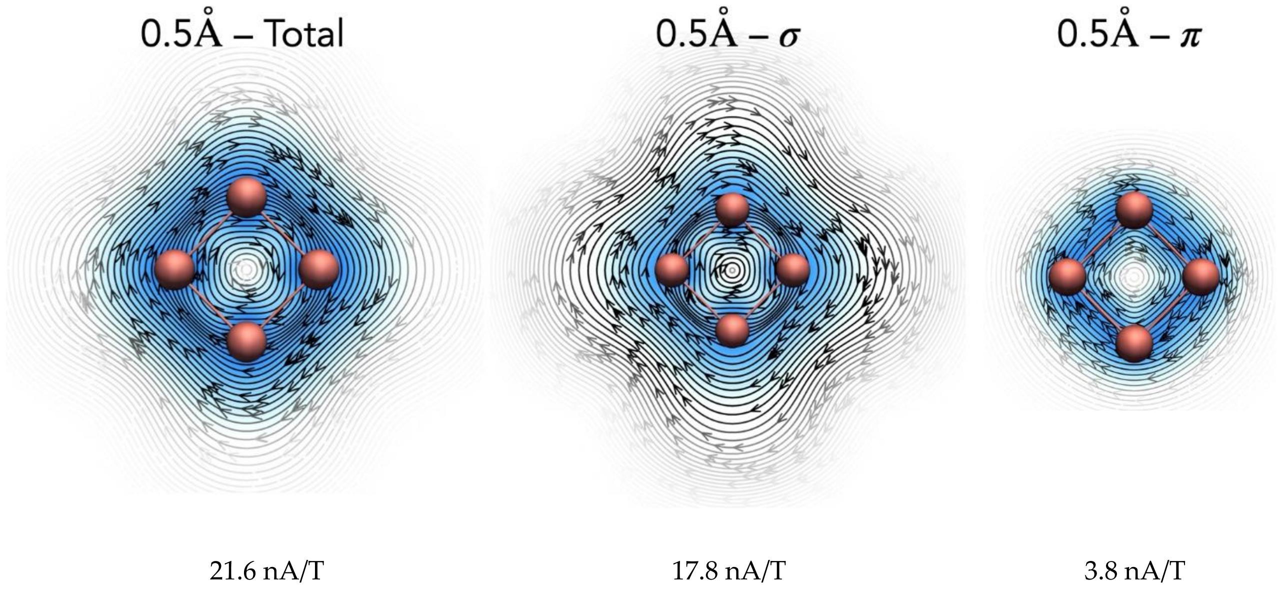

Figure S12. Total, σ- and π-MICD maps 0.5 Å above the molecular plane, with corresponding RCS values of the B_4^{2-}. Calculated at the BHandHLYP/def2-TZVP//PBE0-D3/def2-TZVP level.

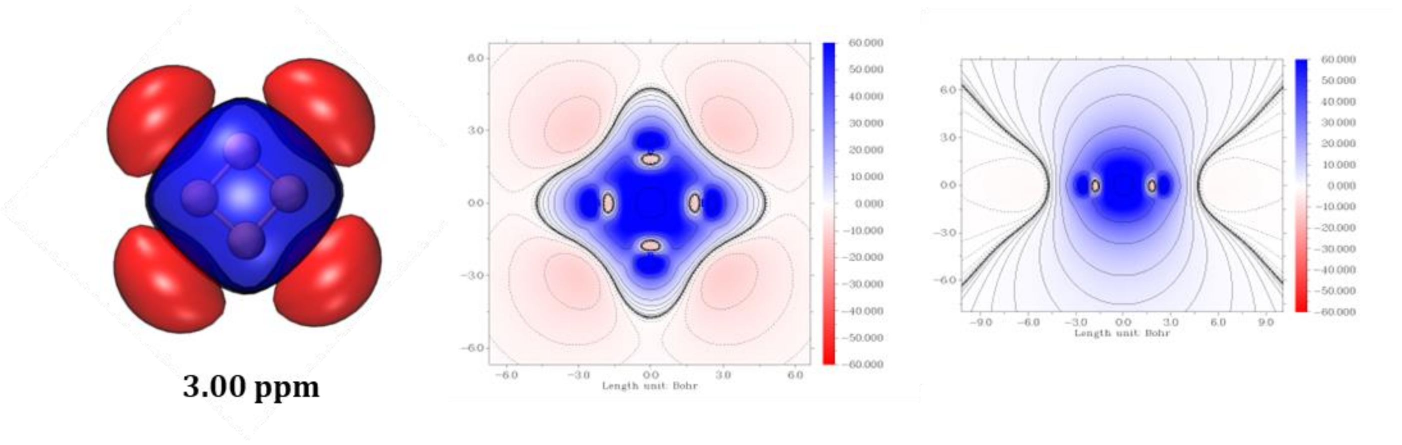

Figure 13. Out-of-plane component of the induced magnetic field (B_z^{ind}), shown as an isosurface (left, isovalue = –3.00 ppm) and 2D contour maps on perpendicular planes of the B_4^{2-} system (center and right). Calculated at the BHandHLYP/def2-TZVP//PBE0-D3/def2-TZVP level of theory. Blue surfaces correspond to regions of magnetic shielding, while red surfaces indicate deshielding.

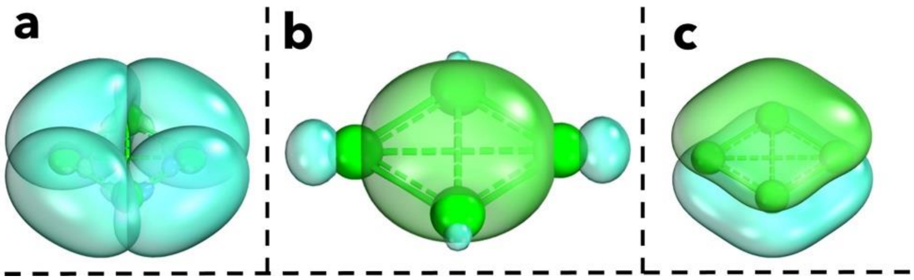

Figure S14. Intrinsic Bond Orbitals (IBOs) of B_4: (a) four tangential σ bonds (2c–2e), (b) one radial σ bond (4c–2e), and (c) one delocalized π bond (4c–2e).

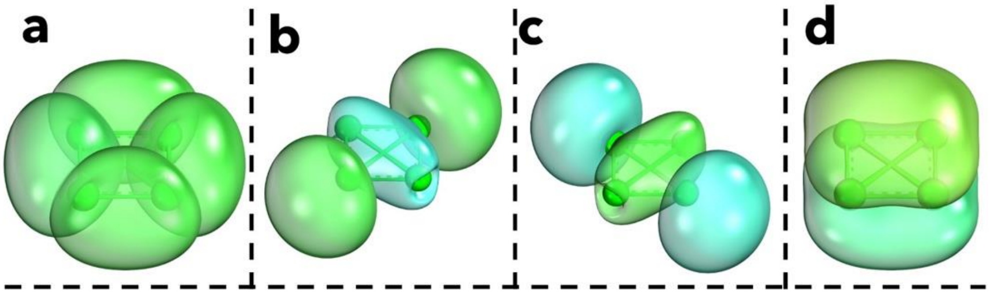

Figure S15. Intrinsic Bond Orbitals (IBOs) of B_4^{2-}: (a) four tangential B_4 σ bonds (2c–2e), (b–c) two radial-tangential mixed σ bonds (4c–2e), and (d) one delocalized π bond (4c–2e).

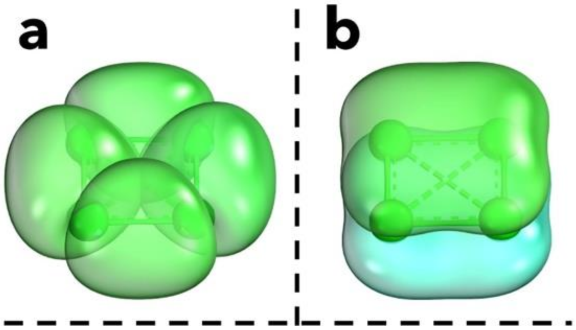

Figure S16. Intrinsic Bond Orbitals (IBOs) of B_4^{2+}: (a) four tangential σ bonds (4c–2e) and (b) one delocalized π bond (4c–2e).

Table S1. Summary of σ- and π-electron delocalization (EDDB values, in electrons), electron counts satisfying the 4n + 2 Hückel rule as derived from AdNDP bonding patterns, and corresponding ring current strengths (in nA/T) from MICD analysis for selected boron clusters and reference hydrocarbons.

**Systems**	**Symmetry**	**EDDB**			**AdNDP analysis**			**Ring Currents Strength**		
		σ	π	Total	σ_{rad}	σ_{tan}	π	$\sigma_{rad+tan}$	π	Total
**B_3^-**	D_{3h}	1.9	1.8	3.7	4n+2	4n+2	4n+2	12.1	3.9	16.0
**B_3^+**	D_{3h}	2.2	1.8	4.0	---	4n+2	4n+2	7.4	3.8	11.2
**B_4**	D_{2h}	1.9	1.5	3.4	4n+2	4n	4n+2	-5.1	3.9	-1.2
**B_4^{2-}**	D_{4h}	1.3	1.5	2.8	4n+2	4n+2	4n+2	17.8	3.8	21.6
**B_4^{2+}**	D_{4h}	0.7	1.5	2.2	---	4n+2	4n+2	-5.7	3.9	-1.8
**C_6H_6**	D_{6h}	0.7	5.3	6.0	---	---	4n+2	0.3	11.9	12.2
**C_4H_4**	D_{2h}	0.3	0.0	0.3	---	---	4n	-5.4	-15.6	-21.0

Cartesian Coordinates

Table S2. Cartesian coordinates of the B_3^-, B_3^+, B_4, B_4^{2+}, and B_4^{2-} optimized structures at the PBE0-D3/def2-TZVP level.

**B_3^-**				**B_3^+**			
5	0.000000000	0.888401000	0.000000000	5	0.000000000	0.900959000	0.000000000
5	0.769378000	-0.444201000	0.000000000	5	0.780253000	-0.450479000	0.000000000
5	-0.769378000	-0.444201000	0.000000000	5	-0.780253000	-0.450479000	0.000000000
**B_4**				**B_4^{2+}**			
5	-1.197288000	0.000000000	0.000000000	5	0.000000000	1.119909000	0.000000000
5	0.000000000	0.938677000	0.000000000	5	0.000000000	-1.119909000	0.000000000
5	0.000000000	-0.938677000	0.000000000	5	1.119909000	0.000000000	0.000000000
5	1.197288000	0.000000000	0.000000000	5	-1.119909000	0.000000000	0.000000000
**B_4^{2-}**							
5	0.000000000	1.163882000	0.000000000				
5	1.163882000	0.000000000	0.000000000				
5	-1.163882000	0.000000000	0.000000000				
5	0.000000000	-1.163882000	0.000000000				
